# Gene cluster analysis for the biosynthesis of elgicins, novel lantibiotics produced by *paenibacillus elgii *B69

**DOI:** 10.1186/1471-2180-12-45

**Published:** 2012-03-26

**Authors:** Yi Teng, Wenpeng Zhao, Chaodong Qian, Ou Li, Liang Zhu, Xuechang Wu

**Affiliations:** 1 Institute of Microbiology, College of Life Sciences, Zhejiang University, 866 Yuhangtang Road, Hangzhou 310058, P. R. China

## Abstract

**Background:**

The recent increase in bacterial resistance to antibiotics has promoted the exploration of novel antibacterial materials. As a result, many researchers are undertaking work to identify new lantibiotics because of their potent antimicrobial activities. The objective of this study was to provide details of a lantibiotic-like gene cluster in *Paenibacillus elgii *B69 and to produce the antibacterial substances coded by this gene cluster based on culture screening.

**Results:**

Analysis of the *P. elgii *B69 genome sequence revealed the presence of a lantibiotic-like gene cluster composed of five open reading frames (*elgT1*, *elgC*, *elgT2*, *elgB*, and *elgA*). Screening of culture extracts for active substances possessing the predicted properties of the encoded product led to the isolation of four novel peptides (elgicins AI, AII, B, and C) with a broad inhibitory spectrum. The molecular weights of these peptides were 4536, 4593, 4706, and 4820 Da, respectively. The N-terminal sequence of elgicin B was Leu-Gly-Asp-Tyr, which corresponded to the partial sequence of the peptide ElgA encoded by *elgA*. Edman degradation suggested that the product elgicin B is derived from ElgA. By correlating the results of electrospray ionization-mass spectrometry analyses of elgicins AI, AII, and C, these peptides are deduced to have originated from the same precursor, ElgA.

**Conclusions:**

A novel lantibiotic-like gene cluster was shown to be present in *P. elgii *B69. Four new lantibiotics with a broad inhibitory spectrum were isolated, and these appear to be promising antibacterial agents.

## Background

Bacteriocins are ribosomally synthesized antibacterial peptides produced by bacteria that possess inhibitory activity against closely related species. Two major types of bacteriocins can be distinguished according to their posttranslational modifications: Class I, the modified bacteriocins or lantibiotics, and Class II, the unmodified bacteriocins. Lantibiotics are a group of small (< 5 kDa) modified bacteriocins characterized by the presence of unusual amino acids such as the thioether-bridge-containing amino acids lanthionine (Lan) and methyl-lanthionine (MeLan), and several dehydrated amino acids such as α,β-didehydroalanine (Dha) and α,β-didehydrobutyrine (Dhb). Most lantibiotics show broad antibacterial activity. For instance, nisin, a safe food preservative [1], displays potent activity against Gram-positive bacteria, including spoilage and pathogenic bacteria such as *Bacillus cereus*, *Listeria monocytogenes*, *Enterococcus*, *Staphylococcus*, and *Streptococcus *[2]. However, some peptides (notably lantipeptides containing Lan and MeLan residues) such as SapB [3] show no antibacterial activity.

Lantibiotics are synthesized as prepeptides that consist of an N-terminal leader sequence and a propeptide part; these prepeptides subsequently undergo posttranslational modifications to become the mature antibiotic. The formation of Lan and MeLan are attributed to the intermolecular cyclization of the thiol groups of cysteine residues with Dha and Dhb, which are obtained from the dehydration of serine and threonine residues, respectively. Dedicated biosynthetic enzymes are required during the process of maturation and the genes encoding these proteins are clustered, as described for nisin [4,5], pep5 [6], nukacin ISK-1 [7], epicidin 280 [8], and mersacidin [9]. According to the genetic organization of lantibiotics, they can be divided into several types [10,11]. The typical gene cluster of type AI lantibiotics, such as nisin and epidermin, includes the structural gene *lanA*, modification enzyme-encoding genes *lanB *and *lanC*, the processing protease-encoding gene *lanP*, the transporter gene *lanT*, and the immunity genes *lanI *and/or *lanEFG*. However, not all type AI lantibiotic-like gene clusters contain all these genes; for example, in the gene cluster *spaBTCAIFGRK *[12], which codes for the biosynthesis of subtilin, the function of LanP is replaced by an intrinsic protease of *Bacillus subtilis *ATCC 6633 [13].

Much attention has been concentrated on the identification of new lantibiotics because of their potent antimicrobial activities. In recent years, with the availability of abundant genomic sequence data in public databases, many new lantibiotics and lantipeptides such as Bsa, lichenicidin, and a range of cyanobacteria-associated lantipeptides [14-16] have been identified. For example, the bacterial genus *Paenibacillus *is known for its ability to produce peptide antibiotics [17-19], and an increasing number of *Paenibacillus *spp. genomes have been sequenced, revealing several novel lantibiotic-related gene clusters [20,21]. However, to date, only one novel lantibiotic, paenibacillin, produced by *Paenibacillus polymyxa *OSY-DF [22] has been reported. In the present study, we present the detailed bioinformatic analysis of a novel lantibiotic-like gene cluster in the *Paenibacillus elgii *B69 genome. Screening of bacterial cultures, mass spectrometry (MS) analysis, and N-terminal amino acid sequencing were used to confirm that the *P. elgii *B69 gene cluster encodes elgicins, novel broad-spectrum lantibiotics.

## Results and discussion

### Putative lantibiotic-like gene cluster of P. Elgii B69

**Figure 1:**
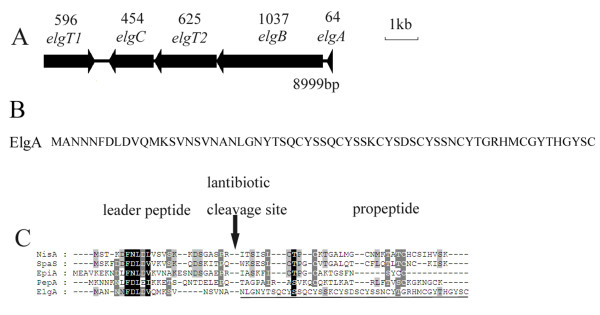
***Elg *gene cluster, ElgA amino acid sequence and sequence alignment with type AI prelantibiotics**. A, The biosynthetic gene cluster of *P. elgii *B69 consists of five ORFs, *elgT1*, *elgC*, *elgT2*, *elgB*, and *elgA*. The number of amino acids encoded by each gene is indicated below each locus, and the arrows indicate the relative directions of transcription. B, The amino acid sequence of the prepeptide ElgA. C, Sequence alignment of the deduced pre-elgicin (ElgA) with type AI prelantibiotics of nisin (NisA), subtilin (SpaS), epidermin (EpiA), and Pep5 (PepA). The conserved residues are shaded and the cleavage sites of the processing protease are symbolized by vertical solid arrows. The resulting propeptide of the cleaved ElgA in the figure is elgicin C (underlined). ElgA is a type AI prelantibiotic because of the conserved motif "FDLD" in its leader peptide segment and the presence of the genes *elgB *and *elgC*.

*P. elgii *B69 was subjected to whole-genome shotgun sequencing, yielding 7.9 Mb of sequence on 278 assembled contigs [23]. Data mining for the LanC homolog amidst the genomic data of *P. elgii *B69, using the SpaC sequence of *P. polymyxa *E681 as a driver, resulted in the identification of a lantibiotic-like gene cluster containing five probable open reading frames (ORFs), designated *elgT1*, *elgC*, *elgT2*, *elgB*, and *elgA *(Figure 1A). All genes, except *elgT1*, were transcribed in the same orientation. The amino acid sequences of the four products of the *elg *gene (*elgT1CT2B*) showed high levels of identity (31%-38%) with those of homologous proteins from several type AI lantibiotic gene clusters (Table 1).

**Table 1:** Deduced peptides and proteins derived from the *elg *gene cluster

ORF	Size of Putative Protein (aa)	Putative Function	Sequence Homolog (GenBank ID)	Identities (%; No. of amino acids)
*elgT1*	596	Transportation and secretion, ABC transporter	Putative SpaT, *Bacillus subtilis *A1/3, AAL15565	31; 614
*elgC*	454	Synthetase in posttranslational modification	Lantibiotic cyclase MibC, *Microbispora corallina *NRRL 30420, ADK32556	36; 485
*elgT2*	625	Transportation and secretion, ABC transporter	Subtilin transport ATP-binding protein SpaT, *Bacillus *subtilis ATCC 6633, P33116	38; 614
*elgB*	1037	Dehydration of serine and threonine	Lantibiotic dehydratase MibB, *Microbispora corallina *NRRL 30420, ADK32555	31; 1115
*elgA*	64	Elgicins	PREDICTED: similar to HECT, C2, and WW domain, containing E3 ubiquitin, XP_001507682	59; 1657

ElgT1 (596 amino acids (a.a.)) and ElgT2 (625 a.a.) showed high-level identity with numerous adenosine-5'-triphosphate (ATP)-binding cassette (ABC) transporter proteins. ElgT1 shared 31% identity with SpaT, a protein responsible for the transportation of the ericins A and S of *B. subtilis *A1/3 [GenBank: AAL15565] [12], and 31% identity with EtnT, which is responsible for the export of the entianin of *B. subtilis *subsp. *spizizenii *DSM 15029^T ^[GenBank: AEK64492] [24]. Similarly, ElgT2 showed strong homology (38% identity) with the subtilin-transport protein of *B. subtilis *ATCC 6633 [GenBank: P33116] [25], and was homologous to NisT of *Lactococcus lactis *N8 [GenBank: CAA79469] and NsuT of *Streptococcus uberis *42 [GenBank: ABA00880] (34% identity in both cases). These proteins are responsible for the transportation of nisin Z and nisin U, respectively [26,27]. The two proteins ElgT1 and ElgT2 also shared 28% sequence identity with each other, suggesting that they have similar functions in the processes of transportation and secretion of elgicins. ElgT1 and ElgT2 may serve as a two-component ABC transporter, similar to MibTU and CinTU, which are probably involved in the export of microbisporicin and cinnamycin [28,29]; however this function is uncommon in the maturation of lantibiotics.

*ElgC *encodes a protein containing 454 amino acids, which shows strong homology to the lantibiotic cyclase, MibC, of *Microbispora corallina *NRRL 30420 (33% identity) [GenBank: ADK32556]. MibC is involved in the formation of (Me)Lan bridges in microbisporicin [28]. The amino acid sequences encoded by the *lanC *genes have some conserved structural motifs, including GXAHG, WCXG, and CHG, in which the cysteine and histidine residues are highly conserved [30]. The alignment of ElgC with several type AI lantibiotic synthetases showed that ElgC contains several conserved sequences, such as GVSHG (positions 244-248), WCYG (positions 316-319), and CHG (positions 366-368), wherein His247, Cys317, Cys366, and His367 are strictly conserved. These observations indicate that ElgC is a lantibiotic synthetase that catalyzes the synthesis of Lan and MeLan residues.

A large ORF upstream and overlapping *elgT2 *by 4 bp encodes a protein of 1037 amino acids. The putative protein ElgB shares 31% identity with MibB of *M. corallina *NRRL 30420 [GenBank: ADK32555] and 30% identity with SpaB of *B. subtilis *ATCC 6633 [GenBank: P39774]. The proteins MibB and SpaB are responsible for the dehydration of serine and threonine residues in the propeptide to form the unsaturated amino acids of microbisporicin and subtilin, respectively [28,31]. Thus, ElgB appears to be a dehydratase involved in the process of maturation.

Similarly, *elgA *encodes the prepeptide of the elgicins, with a length of 64 amino acids. No lantibiotics reported thus far share homology with ElgA, suggesting that the mature proteins derived from ElgA are novel lantibiotics. The alignment of the putative leader peptide of ElgA with those of other lantibiotics revealed the existence of a possibly conserved motif "FDLD" (Figure 1C), which resembles the "FDLN" motif in the leader peptide of type AI lantibiotics [32]. Considering that the *elg *gene cluster contains the *lanB *and *lanC *genes encoding the modified enzymes, it could be concluded that the elgicins are type AI lantibiotics.

The *elg *gene cluster lacks the immunity genes *lanI *and *lanEFG*. LanEFG acts as an ABC transporter for lantibiotic immunity; for example, NisEFG expels lantibiotic molecules that have entered the cytoplasmic membrane into the extracellular environment [33]. Considering the mechanism of LanEFG-imparted immunity, ElgT1T2 is likely to play a role in self-protection, in addition to that of secretion and transportation of the elgicins.

The leader peptides of type AI lantibiotics are usually processed by a serine protease encoded by *lanP*, which is not found in the *elg *gene cluster. The leader peptide of ElgA may instead be processed by an intrinsic B69 serine protease. This elgicin maturation process might therefore resemble that of subtilin, wherein the leader peptide of presubtilin is removed by an intrinsic *B. subtilis *subtilisin-like protease [13].

### Isolation and purification of elgicins

Genomic analysis of *P. elgii *B69 revealed the presence of a new lantibiotic-like gene cluster. To express this *elg *gene cluster, *P. elgii *B69 was grown aerobically at 30°C for 120 h in different fermentation media designed for the production of active substances. At harvest, extractions of B69 fermentation broths were achieved using column chromatographic fractionation on AB-8 macroporous resin (Haiguang Chemical Ltd., Tianjin, China). The KL medium fraction (5 g/L glucose, 4 g/L (NH_4_)_2_SO_4_, 2.6 g/L K_2_HPO_4_, 4 g/L MgSO_4_, 2 g/L NaCl, 2 g/L CaCl_2_, 2 mg/L FeSO_4_·7H_2_O, 2 mg/L ZnSO_4_·7H_2_O, and 1.5 mg/L MnSO_4_·H_2_O, pH 7.2) eluted by 80% methanol showed activity against the indicator strain *P. ehimensis*. This fraction was then applied to the solid-phase extraction (SPE) column. The fraction with activity against the indicator strain was eluted with 50% methanol and further separated by analytical reverse-phase high-performance liquid chromatography (RP-HPLC). Aided by the presence of several tyrosine residues within the precursor peptide ElgA, its ultraviolet (UV) absorption was measured at 280 nm during analytical HPLC. The fractions corresponding to the retention time of 21.290-22.036 min were isolated, and they showed activity against *P. ehimensis*.

**Figure 2:**
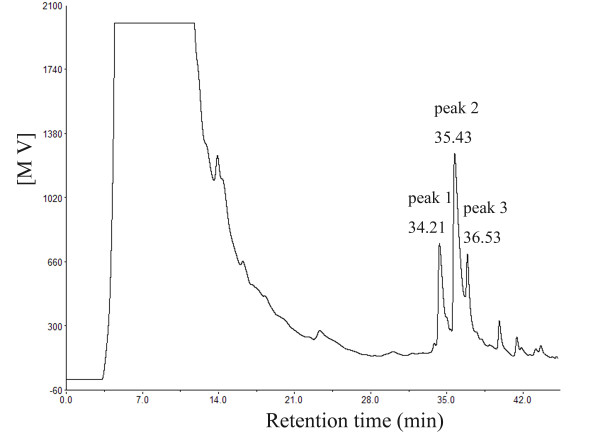
**Reverse-phase HPLC profile of crude SPE-extraction**. UV absorption was measured at 280 nm. MV, millivolt. Peak 1, with retention time of 34.21 min, corresponds to elgicins AI and AII. Peaks 2 and 3, with retention times of 35.43 and 36.53 min, correspond to elgicins B and C, respectively.

Large-scale fermentation of *P. elgii *B69 was carried out in KL medium for the production of active substances. The target compounds were then isolated by a simple three-step purification procedure consisting of AB-8 resin fractionation, SPE, and preparative RP-HPLC, as described in the "Methods" section. In the preparative RP-HPLC profile, the three peaks corresponding to retention times of 34.21, 35.43, and 36.53 min (Figure 2) were pooled and designated elgicin A, B, and C, respectively, of which elgicin B was the major component. These fractions were lyophilized and subjected to electrospray ionization-mass spectrometry (ESI-MS) for molecular analyses.

### ESI-MS analyses of elgicins

**Figure 3:**
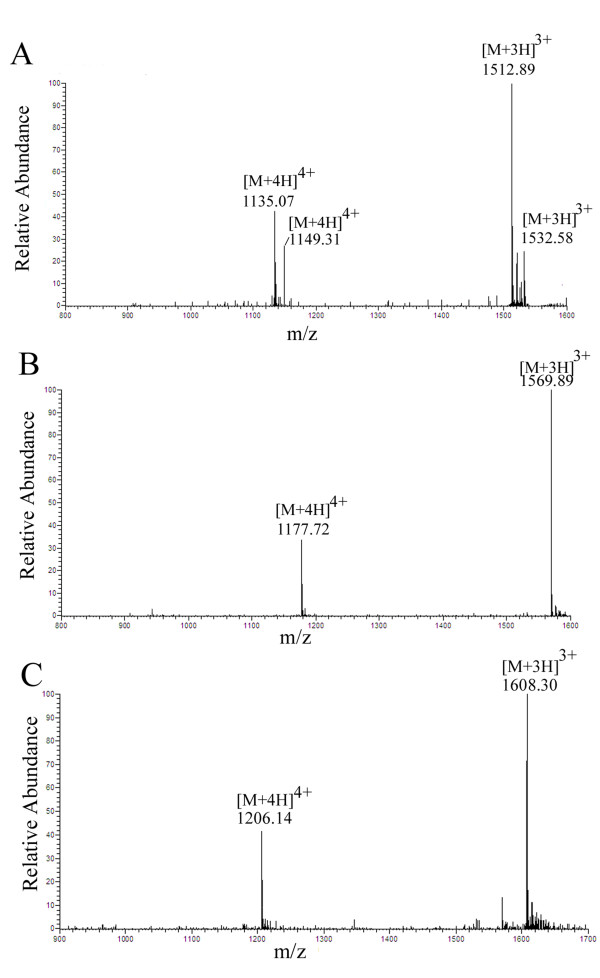
**ESI-MS of RP-HPLC-purified elgicins AI, AII, B, and C isolated from fermentation medium**. A, Peaks at 1512.89 [M + 3H]^3+ ^and 1135.07 [M + 4H]^4+ ^correspond to a mass of 4536 Da for elgicin AI. Peaks at 1532.58 [M + 3H]^3+ ^and 1149.31 [M + 4H]^4+ ^correspond to a mass of 4593 Da for elgicin AII, indicating that it has one Gly residue more than elgicin AI. B, Peaks at 1569.89 [M + 3H]^3+ ^and 1177.72 [M + 4H]^4+ ^correspond to a mass of 4706 Da for elgicin B, indicating that it has one Leu residue more than elgicin AII. C, Peaks at 1608.30 [M + 3H]^3+ ^and 1206.14 [M + 4H]^4+ ^correspond to a mass of 4820 Da for elgicin C, indicating that it has one Asn residue more than elgicin B.

To determine the molecular masses of elgicins, the lyophilized elgicins A, B, and C were dissolved in sterile water and subjected to ESI-MS. The MS spectrum of HPLC-purified elgicin A revealed four signals at the mass-to-charge ratios (*m/z*) 1135.07 [M + 4H]^4+^, 1512.89 [M + 3H]^3+^, 1149.31 [M + 4H]^4+^, and 1532.58 [M + 3H]^3+ ^(Figure 3A). The molecular weight calculated from the two former signals was 4536 Da, and the others corresponded to a molecular weight of 4593 Da. These findings suggest that Peak 1 contained two compounds, designated elgicin AI and elgicin AII. The molecular weight of elgicin AII was 57 Da larger than that of elgicin AI; this difference corresponds to the molecular weight of a single glycine residue. In the case of Peak 2, the mass spectrum showed the presence of two strong signals at *m/z *values of 1177.72 [M + 4H]^4+ ^and 1569.89 [M + 3H]^3+^, corresponding to a molecular mass of 4706 Da (Figure 3B). The molecular weight of elgicin B was 113 Da larger than that of AII; this difference corresponds to the molecular mass of a single leucine residue, as deduced from the prepeptide of ElgA that lacks an isoleucine residue (Figure 1B). Compound elgicin C, with a retention time of 36.53 min, had a molecular mass of 4820 Da, consistent with the two signals at *m/z *1206.14 [M + 4H]^4+ ^and 1608.30 [M + 3H]^3+ ^(Figure 3C). The molecular mass of elgicin C was 114 Da larger than that of elgicin B; this difference is consistent with the molecular mass of a single asparagine residue.

Lantibiotics have small molecular weights (< 5 kDa) that usually range from 1700-4000 Da. Thus far, the molecular weights of only two lantibiotics, cytolysin L_L _(isolated from the *Enterococcus faecalis *strain FA2-2) and carnocin U149 (produced by *Carnobacterium piscicola *U149), exceed 4 kDa (4164 and 4635 Da, respectively) [10]. Our newly isolated four-component elgicins therefore have unusually large molecular weights of 4536 Da (elgicin AI), 4593 Da (elgicin AII), 4706 Da (elgicin B), and 4820 Da (elgicin C). To the best of our knowledge, no other lantibiotics have molecular weights greater than those of elgicins B and C.

### Analysis of N-terminal amino acid sequence

**Figure 4:**
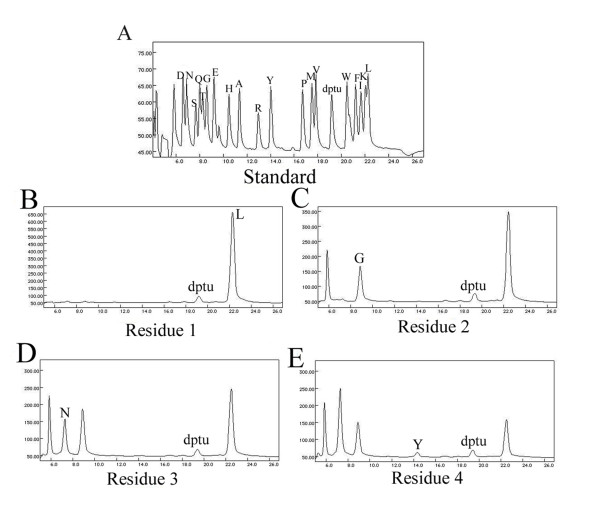
**Determination of N-terminal sequence of elgicin B using standard Edman degradation method**. A, The 20 known amino acids served as standards. The peak representing the cysteine residue was not labeled. B-E, The first four amino acids in the N-terminal region of elgicin B (leucine, glycine, asparagine, and tyrosine) were determined. Diphenylthiourea (*dptu*) is the by-product of the Edman degradation reaction.

To confirm whether the four-component antibacterial agents are derived from ElgA, HPLC-purified elgicin B was subjected to automated Edman degradation to determine its N-terminal amino acid sequence (Figure 4). The first four amino acids were Leu-Gly-Asp-Tyr. The fifth residue was blocked completely, suggesting the presence of a dehydrated amino acid residue, a characteristic feature of lantibiotics. The Leu-Gly-Asp-Tyr sequence was consistent with the sequence of the propeptide that resulted from the removal of the leader peptide after cleavage at positions ranging between Asp21 and Leu22 of ElgA (Figure 1B). The observed molecular weight of elgicin B was 144 Da smaller than the calculated molecular weight of the unmodified propeptide, which can be explained by the loss of eight H_2_O molecules during posttranslational modification. Therefore, elgicin B is deduced to be the posttranslational modified product of ElgA.

The residue at position 21 of ElgA (Figure 1B) was asparagine and leucine was found at position 22. Considering the ESI-MS results, wherein the molecular weight of elgicin C was 114 Da larger and that of elgicin AII 113 Da smaller than that of elgicin B, the N-terminal amino acid sequences of the unmodified propeptides of elgicins C and AII could be Asp-Leu-Gly-Asp-Tyr and Gly-Asp-Tyr, respectively. Similarly, because the glycine residue was at position 23 of ElgA and the molecular weight of elgicin AI was 57 Da smaller than that of elgicin AII, the N-terminal amino acid sequence of the unmodified propeptide of elgicin AI could be Asp-Tyr. The observed molecular weights of these three peptides were 144 Da smaller than the calculated molecular weights of the respective predicted propeptides. This finding may be attributed to the loss of eight H_2_O molecules during maturation. Elgicins AI, AII, and C were thus confirmed to be the modified products of ElgA, that is, these four antibacterial agents possibly originated from the same prepeptide, ElgA, by peptide cleavage, followed by the removal of one amino acid at each N-terminal.

In the *elg *gene cluster, the presence of *elgB, elgC*, and the leader peptide of ElgA containing the motif "FDLD" confirmed that the elgicins are type AI lantibiotics. The origin of elgicins from identical pre-peptides by peptide cleavage and the removal of one amino acid from each corresponding N-terminus could be achieved in two ways. First, the serine protease could cleave at four cleavage sites of ElgA, that is, Ala20-Asp21, Asp21-Leu22, Leu22-Gly23, and Gly23-Asp24 (Figure 1B), resulting in the simultaneous production of these four peptides. Second, the Ala20-Asp21 could be cleaved by the serine protease to produce elgicin C, followed by the successive protease removal of Asp21, Leu22, or Gly23 residues from elgicin C to yield elgicins B, AII, and AI, respectively.

### Antimicrobial activity of elgicins

Preparative RP-HPLC-purified elgicin compounds (150 μg) were pipetted onto a sterile paper disk and tested for antibacterial activity against various bacterial strains. As shown in Table 2, the active substances produced by *P. elgii *B69 showed inhibitory activity toward *Staphylococcus epidermidis *CMCC 26069, *Staphylococcus aureus *ATCC 43300, *Pseudomonas aeruginosa *ATCC 27853, *Escherichia coli *ATCC 35218, and *Proteus vulgaris *CMCC 49027. Other tested strains, namely, *S. aureus *ATCC 25923 and *B. subtilis *CGMCC 1.1470, were resistant to elgicins.

**Table 2:** Antibacterial spectra of RP-HPLC-purified elgicin compounds

Indicator Strain	Diameter of Inhibition (mm)	
	**Elgicins**^**a**^	**Polymyxin B**^**b**^
*Staphyloccus epidermidis *CMCC 26069	8	18
*Staphylococcus aureus *ATCC 43300	8	15
*Staphylococcus aureus *ATCC 25923	0	0
*Bacillus subtilis *CGMCC 1.1470	0	10
*Pseudomonas aeruginosa *ATCC 27853	7	12
*Escherichia coli *ATCC 35218	9	10
*Proteus vulgaris *CMCC 49027	8	0

^a^The amount of elgicins is 150 μg per disk.

^b^The amount of polymyxin B is 30 μg per disk.

## Conclusions

Genomic sequence analysis of *Paenibacillus elgii *B69 showed a novel lantibiotic-like gene cluster. Four new lantibiotics, designated elgicins AI, AII, B, and C, were isolated from the KL medium. To the best of our knowledge, elgicins B and C are the largest reported lantibiotics to date, with molecular weights of 4706 and 4820 Da, respectively. Elgicins have broad inhibitory activities against several Gram-positive and Gram-negative bacteria. Further studies are required to determine their structures, identify their mechanisms of action, and find suitable bioprocessing strategies for efficient elgicin production.

## Methods

### Bacteria and culture conditions

*P. elgii *B69 was isolated from a soil sample collected from Hangzhou, China [19]. Nutrient broth was routinely used for culturing *P. elgii *B69 at 30°C for 24 h. The active substances were produced in synthetic medium (KL). About 25 mL of the *P. elgii *B69 culture was used to inoculate 2-L conical flasks, each containing 500 mL of KL medium. Four other fermentation media, Landy medium (20 g/L glucose, 5 g/L L-glutamic acid, 0.5 g/L MgSO_4_, 0.5 g/L KCl, 1 g/L KH_2_PO_4_, 0.15 mg/L Fe(SO_4_)_3_·6H_2_O, 5.0 mg/L MnSO_4_·H_2_O, and 0.16 mg/L CuSO_4_·5H_2_O) [34], MYPGP broth (15 g/L yeast extract, 10 g/L Mueller-Hinton broth, 2 g/L glucose, 3 g/L K_2_HPO_4_, and 1 g/L sodium pyruvate) [35], AK medium (0.5 g/L asparagine, 0.5 g/L K_2_HPO_4_, 0.2 g/L MgSO_4_, 0.01 g/L FeSO_4_·7H_2_O, and 10 g/L glucose), and Luria-Bertani (LB) medium, were used to test for the presence of inhibitory factors.

The fermentation batches were incubated aerobically on a shaker (200 rpm) at 30°C for 120 h. The test strains used to determine sensitivity to elgicins included *S. epidermidis *CMCC 26069, *S. aureus *ATCC 43300, *S. aureus *ATCC 25923, *B. subtilis *CGMCC 1.1470, *P. aeruginosa *ATCC 27853, *E. coli *ATCC 35218, and *P. vulgaris *CMCC 49027. *P. ehimensis*, a closely related species of *P. elgii*, was used as the indicator strain. All test stains were grown in nutrient broth or nutrient agar plates at 37°C. For stock preparation, the cells were cultivated for 24 h, mixed with sterile glycerol (to a final concentration of 25%, v/v), and stored at -80°C.

### Bioinformatic analyses

Using the modification enzyme SpaC of *P. polymyxa *E681 [GenBank: YP_003869828] as a driver sequence, the draft genome sequence of *P. elgii *B69 was examined for homology using the basic local alignment search tool (BLAST). The ORFs of the gene cluster were identified using an ORF finder http://www.ncbi.nlm.nih.gov/gorf/gorf.html. Amino acid sequence identities of the proteins were identified by searching the National Center for Biotechnology Information (NCBI) database using BLAST. Alignment was carried out using MEGA 4.0.1 software [36].

### Isolation and purification of elgicins

Stationary-phase cells were removed from the 3-L fermentation medium by centrifugation at 5000 rpm for 30 min at 4°C. The cell-free supernatant was loaded onto an AB-8 macroporous absorption resin column preequilibrated with distilled water. The column was washed sequentially with distilled water, followed by elution with 20% and 80% (v/v) methanol. All fractions, except those eluted with 80% methanol, were discarded. The 80% methanol fraction was pooled and concentrated at 45°C using a rotary evaporator. The resulting contents, which totaled approximately 70 mL, were centrifuged at 7000 rpm for 30 min at 4°C. The supernatant was applied to a C_18 _SPE column (Hardwee, Germany) pretreated with distilled water. The column was washed with three bed volumes of distilled water, followed by three bed volumes of 30% methanol. These fractions were discarded. The fraction containing the active substances was recovered from the column by washing with two bed volumes of 50% methanol and concentrated by vacuum evaporation at 45°C.

Aliquots (12 mL) of this material were further separated by preparative reverse-phase high-pressure liquid chromatography (RP-HPLC), in a system equipped with a YMC-pack ODS-A C_18 _(5 μm, 250 mm × 20 mm) column. Eluent A was MilliQ-purified water containing 0.02% trifluoroacetic acid. Acetonitrile was selected as eluent B. Elution was carried out at a flow rate of 10 mL/min using a constant gradient of 20% eluent B for 15 min, followed by a linear gradient of eluent B ranging from 20-35% over a period of 30 min. The process was detected spectrophotometrically by measuring the absorption values at 280 nm. The fractions containing the elgicins were collected, concentrated, and lyophilized to give 12 mg of product, which was dissolved in sterile water (0.8 ml) at a concentration of 15 mg/ml.

### Mass spectra and N-terminal amino acid sequence analyses

The molecular weights of the purified elgicins were determined by ESI-MS on a Thermo Finnigan LCQ DECA XP MAX instrument (Thermo Electron Corporation, San Jose, CA). The electrospray source was operated at a capillary voltage of 17.49 V, a source voltage of 4.53 KV, and a capillary temperature of 275.10°C. The mass spectra were measured in the range of 500-2000 *m/z *and analyzed using Xcalibur 1.4 software (Thermo Electron Corporation). The N-terminal amino acid sequence of the purified elgicin B was determined by an automatic sequence analyzer (Gene Core Biotechnologies Co., Ltd., Shanghai, China) using the standard Edman degradation method.

### Assay of antimicrobial activity using the paper disk method

The preparative RP-HPLC-purified elgicin compounds were tested to determine their inhibitory spectra by the paper disk diffusion method. Aliquots of overnight-cultured test strains (100 μL) were spread using a glass rod spreader on nutrient agar plates containing 2% agar. Aliquots (10 μL) of the elgicin compounds were pipetted onto sterilized filter paper disks (0.6 cm in diameter), which were then allowed to dry in an open 9-cm sterile Petri dish at room temperature. The disks were placed on the surface of the inoculated plates and incubated for 18 h at 37°C. The diameters of the zone of inhibition were measured. All analyses were conducted independently in triplicate.

### Nucleotide sequence accession number

The complete nucleotide sequence of the *elg *gene cluster derived in the present study was deposited in the database of the National Center for Biotechnology Information under accession number JQ429086.

## Authors' contributions

XCW and YT envisaged the study and designed the experiments. YT wrote the manuscript and carried out the bioinformatic analysis. YT and WPZ carried out the isolation and purification of the sample, and assayed antibacterial activity. CDQ participated in the design of the study. XCW, OL, and LZ helped to revise the manuscript. All authors read and approved the final manuscript.
