# Correction: gene cluster analysis for the biosynthesis of elgicins, novel lantibiotics produced by *Paenibacillus elgii* B69

**DOI:** 10.1186/1471-2180-12-242

**Published:** 2012-10-24

**Authors:** Yi Teng, Wenpeng Zhao, Chaodong Qian, Ou Li, Liang Zhu, Xuechang Wu

**Affiliations:** 1 Institute of Microbiology, College of Life Sciences, Zhejiang University, 866 Yuhangtang Road, Hangzhou, 310058, P. R. China

## Correction

It has come to our attention that we have used Asp, rather than the correct annotation of Asn, to indicate Asparagine throughout the text [1].

In the abstract this is corrected to: The N-terminal sequence of elgicin B was Leu-Gly-Asn-Tyr, which corresponded to the partial sequence of the peptide ElgA encoded by *elgA*.

In the Results section, subsection ‘Analysis of N-terminal amino acid sequence’, all instances of Asp should be replaced with Asn.

We regret any inconvenience that this inaccuracy in the text might have caused.

## References

[B1] Yi T Wenpeng Z Chaodong Q Ou L Liang Z Xuechang W Gene cluster analysis for the biosynthesis of elgicins, novel lantibiotics produced by Paenibacillus elgii B69 BMC Microbiol 2012 12 45 10.1186/1471-2180-12-45 22443157 PMC3337247

